# Frailty Trajectories Following Adjuvant Chemotherapy and Mortality in Older Women With Breast Cancer

**DOI:** 10.1001/jamanetworkopen.2025.0614

**Published:** 2025-03-12

**Authors:** Emilie D. Duchesneau, Dae Hyun Kim, Til Stürmer, Qoua Her, Zhang Zhang, Nicholas M. Pajewski, Heidi D. Klepin, Kathryn E. Callahan, Jennifer L. Lund

**Affiliations:** 1 Division of Public Health Sciences, Department of Epidemiology and Prevention, Wake Forest University School of Medicine, Winston-Salem, North Carolina; 2 Hinda and Arthur Marcus Institute for Aging Research, Hebrew SeniorLife, Harvard Medical School, Boston, Massachusetts; 3 Department of Epidemiology, Gillings School of Global Public Health, University of North Carolina at Chapel Hill, Chapel Hill; 4 Department of Health Policy and Management, Gillings School of Global Public Health, University of North Carolina at Chapel Hill, Chapel Hill; 5 Division of Public Health Sciences, Department of Biostatistics and Data Science, Wake Forest University School of Medicine, Winston-Salem, North Carolina; 6 Section on Hematology and Oncology, Department of Internal Medicine, Wake Forest University School of Medicine, Winston-Salem, North Carolina; 7 Section on Gerontology and Geriatric Medicine, Department of Internal Medicine, Wake Forest University School of Medicine, Winston-Salem, North Carolina

## Abstract

**Importance:**

Frailty assessed at a single time point is associated with mortality in older women with breast cancer. Little is known about how changes in frailty following cancer treatment initiation affect mortality.

**Objective:**

To evaluate the association between claims-based frailty trajectories following adjuvant chemotherapy initiation and 5-year mortality in older women with stage I to III breast cancer.

**Design, Setting, and Participants:**

This longitudinal cohort study used the Surveillance, Epidemiology, and End Results cancer registries linked to Medicare claims data (claims from 2003-2019). Women aged 65 years or older with stage I to III breast cancer diagnosed from 2004 to 2017 were included. Eligible women underwent breast surgery followed by adjuvant chemotherapy as initial treatment. A landmark design was used to identify frailty trajectories during the year following chemotherapy initiation. Continuous enrollment in Medicare fee-for-service from 180 days before cancer diagnosis through 360 days following chemotherapy initiation (landmark) was required. Women who died or disenrolled before the landmark were excluded. Analyses were conducted between September 2022 and March 2024.

**Exposures:**

Claims-based frailty trajectories during the 360 days following chemotherapy initiation were identified using the Faurot frailty index, a validated claims-based proxy for frailty based on demographics and diagnosis, procedure, and durable medical equipment claims. The Faurot frailty index was calculated every 30 days from chemotherapy initiation through the landmark (360 days after chemotherapy initiation). Claims-based frailty trajectory clusters were identified using longitudinal K-means clustering.

**Main Outcomes and Measures:**

Associations between the claims-based frailty trajectory clusters and 5-year mortality from the landmark were estimated using Kaplan-Meier analysis.

**Results:**

In total, 20 292 women with breast cancer (median [IQR] age, 70 [67-74] years) were identified. The K-means analysis resulted in 6 trajectory clusters: 3 robust (16 120 women [79.4%]) or resilient (3259 [16.1%]) trajectories and 3 nonresilient trajectories (913 women [4.5%]). Five-year mortality was higher in women belonging to the 3 nonresilient trajectories compared with those belonging to the 3 resilient trajectories (52.1% vs 20.3%; difference, 31.8%; 95% CI, 29.0%-36.2%).

**Conclusions and Relevance:**

In this cohort study of women with stage I to III breast cancer, frailty changes following chemotherapy initiation were associated with long-term survival. Future research should assess the association of frailty interventions following cancer treatment initiation with survival and patient-centered outcomes in this population.

## Key Points

**Question:**

Are claims-based frailty trajectories following adjuvant chemotherapy initiation associated with 5-year mortality in older women with breast cancer?

**Findings:**

In this cohort study, among 20 292 women (aged ≥65 years) with stage I to III breast cancer receiving adjuvant chemotherapy, 5-year mortality was higher in women with rapid frailty progression (nonresilience) compared with those with robust (stable) or resilient (transient worsening) frailty trajectories.

**Meaning:**

These findings suggest that frailty trajectories following chemotherapy are associated with long-term survival in older women with breast cancer; future research should explore whether frailty monitoring and interventions improve outcomes in this population.

## Teaser

This cohort study examines the association between frailty trajectories following adjuvant chemotherapy initiation and 5-year mortality in older women with breast cancer.

## Introduction

Breast cancer is the most common cancer in the US, with a median age at diagnosis of 62 years.^1,2^ Frailty is an important prognostic indicator in older women with breast cancer.^3^ Those who are prefrail or frail at treatment initiation are more likely to report high-grade chemotherapy toxic effects, discontinue treatment, report worse health-related quality of life, or die compared with robust counterparts.^4,5,6^

Breast cancer and its treatments may contribute to changes in frailty in older women with breast cancer. Many women experience clinically meaningful, transient worsening in frailty following chemotherapy initiation, followed by long-term recovery (ie, resilience).^7,8^ However, for some, the progression of frailty persists after chemotherapy completion. Although prior studies^5,9^ have shown that baseline frailty is associated with outcomes in older women with breast cancer, there is limited information on how changes in frailty during treatment affect long-term prognosis. Understanding these associations may help develop interventions to manage frailty progression in this population.

We previously identified claims-based frailty trajectories and risk factors for nonresilient trajectories in older women with stage I to III breast cancer receiving adjuvant chemotherapy.^10^ This study evaluated associations between these frailty trajectories and 5-year mortality. We hypothesized that women who experienced rapid frailty progression (nonresilience) following adjuvant chemotherapy initiation would have higher mortality than those with robust (stable) or resilient (transient worsening followed by recovery) frailty trajectories.

## Methods

### Data Source, Study Design, and Population

We conducted a longitudinal cohort study using linked Surveillance, Epidemiology, and End Results (SEER) cancer registries and Medicare claims and enrollment data. The Office of Human Research Ethics of the University of North Carolina at Chapel Hill granted expedited approval for the project and granted a waiver of informed consent since this was a secondary analysis of a limited dataset, in accordance with 45 CFR §46.116(d). This study followed Strengthening the Reporting of Observational Studies in Epidemiology (STROBE) reporting guidelines.^11,12^

**Figure 1.**
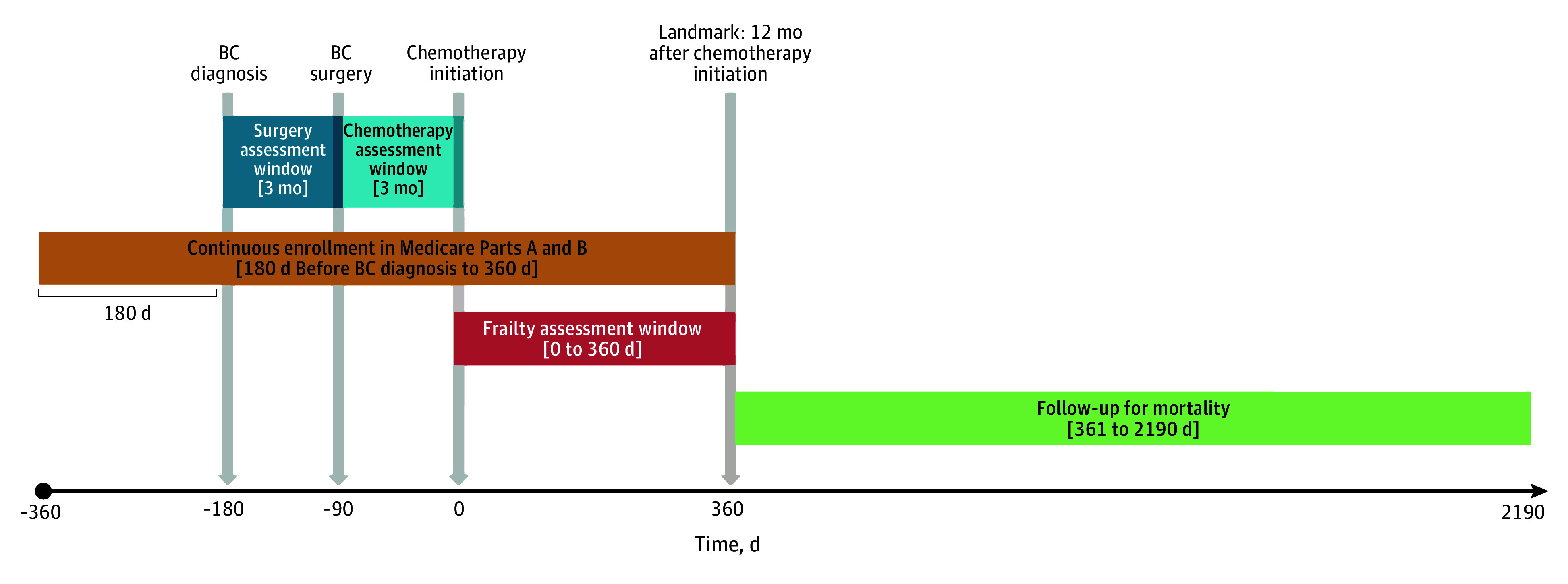
Study Schematic Brackets indicate time intervals relative to chemotherapy initiation (day 0) during which study eligibility criteria and variable definitions were applied. Square brackets indicate that the end points are included in the interval. BC indicates breast cancer.

The study cohort has been described previously^10^ and included older women (aged ≥65 years) with stage I to III breast cancer diagnosed between 2004 and 2017. Medicare claims were available from 2003 to 2019. Eligible women underwent breast cancer surgery (mastectomy or breast conserving therapy) within 90 days of diagnosis and commenced adjuvant chemotherapy within 90 days after surgery (Figure 1).^13^ Women who received a diagnosis of another primary cancer within 180 days before the breast cancer diagnosis, those who received neoadjuvant chemotherapy, and those with metastatic disease at diagnosis were excluded.

We conducted a landmark analysis to identify frailty trajectories during the year following adjuvant chemotherapy initiation (Figure 1).^14,15^ In a landmark analysis, follow-up for an outcome begins at a landmark after a window during which health status or exposures are assessed. In our study, the landmark occurred 360 days following adjuvant chemotherapy initiation. During the window between chemotherapy initiation and the landmark (days 0-360), we assessed claims-based frailty and assigned women to trajectory patterns (details on methods below). Follow-up for mortality began immediately following the landmark (ie, 361 days following chemotherapy initiation). To implement the landmark approach, women were required to be continuously enrolled in Medicare fee-for-service (Parts A and B) with no health maintenance organization enrollment from chemotherapy initiation through the landmark. Women who died or disenrolled from Medicare fee-for-service before the landmark were excluded. We also required continuous enrollment from 180 days prior to the month of breast cancer diagnosis to identify baseline frailty. We followed women from the landmark for up to 5 years to assess mortality.

### Frailty Trajectory Assessment

Women were assigned to 1 of 6 claims-based frailty trajectory clusters according to a prior analysis of this cohort.^10^ The prior analysis used the Faurot frailty index, a validated claims-based proxy measure that calculates a predicted probability of frailty using 20 indicators based on demographic information and claims for diagnosis code groups, procedures, and durable medical equipment during a 6-month lookback period (eTable 1 in Supplement 1).^16,17,18^ The Faurot frailty index was calculated every 30 days from adjuvant chemotherapy initiation to the landmark (day 360). Frailty trajectory clusters were identified using longitudinal K-means clustering, a nonparametric clustering method that identifies optimal clusters by minimizing within group variation and maximizing between group variation.^19,20,21^ The prior analysis identified 1 robust trajectory (low frailty throughout the frailty trajectory assessment window), 2 resilient frailty trajectories (increasing frailty during the initial treatment period, followed by returns to baseline at the end of the frailty trajectory assessment window), and 3 nonresilient frailty trajectories (increasing frailty without a return to baseline during the frailty trajectory assessment window).

### Mortality Assessment

Mortality during the 5-year follow-up period was assessed using vital status and date of death information from the Medicare enrollment file. Follow-up time was administratively censored at the end of data availability (December 31, 2019).

### Covariate Assessment

We measured covariates to describe our study population and for model adjustment. These included age at chemotherapy initiation, race (using the SEER Race Recode variable: American Indian or Alaska Native, Asian or Pacific Islander, Black, White, or unknown),^22^ Hispanic ethnicity (using the SEER Origin Recode variable),^22^ census region, year of cancer diagnosis, the Gagne Combined Comorbidity Score,^23,24^ and tumor characteristics (stage, breast surgery type, tumor size, lymph node involvement, grade, and subtype). The primary sources of race and ethnicity information in SEER are extracted medical records, which may not always reflect self-identified race and ethnicity.^25^ Race and ethnicity were conceptualized as distinct social constructs that influence frailty and mortality, primarily due to systemic racism and inequitable health care access. They were included as covariates to account for the impact of structural and social determinants of health, rather than biological determinants. The Gagne Combined Comorbidity Score was calculated using claims during the 360 days following chemotherapy initiation, excluding cancer and metastatic cancer.

### Subgroup Analyses

We conducted subgroup analyses to assess whether associations between claims-based frailty trajectories and 5-year mortality varied by age and tumor characteristics. We stratified our cohort into subgroups defined by age at chemotherapy initiation (65-74 vs ≥75 years), tumor stage (I, II, or III), and tumor subtype (hormone receptor [HR]–positive and human epidermal growth factor receptor 2 [HER2]–positive, HR-positive and HER2-negative, HR-negative and HER2-positive, or HR-negative and HER2-negative). Since HER2 status was only reliably captured in SEER beginning in 2010, the tumor subtype analysis was restricted to women who received a diagnosis after 2010. We consolidated the 6 trajectories into resilient and nonresilient groups in the analyses of tumor stage and subtype, due to sample size constraints.

### Statistical Analysis

 Analyses were performed between September 2022 and March 2024 using SAS statistical software version 9.4 (SAS Institute) and R statistical software version 4.2.3 (R Project for Statistical Computing). We described the distribution of covariates in the study population, overall and stratified by the frailty trajectories. We described changes in the prevalence of the frailty indicators between chemotherapy initiation and the landmark, consolidating the 6 trajectories into resilient and nonresilient groups due to sample size constraints.

We estimated the cumulative incidence (risk) of 5-year mortality from the landmark using unadjusted Kaplan-Meier analysis, stratifying by the 6 claims-based frailty trajectory clusters. Differences in 5-year mortality risk between the frailty trajectories were calculated using the robust trajectory as the reference group. We estimated 95% CIs using the 2.5th and 97.5th percentiles from 2000 bootstrapped samples.

We compared 3 Cox proportional hazards models to determine whether claims-based frailty trajectories were predictive of 5-year mortality. The first model included the patient demographic and clinical covariates and baseline frailty score (calculated at chemotherapy initiation) as predictors. The second model included the same predictors as the first model and a categorical variable indicating trajectory cluster assignment. On the basis of our findings, we conducted a third ad hoc Cox model that included the covariates, the baseline frailty score, and the final frailty score calculated on the landmark date (ie, 360 days following chemotherapy initiation) as predictors. We compared discrimination across each model using the Harrell C-index.^26^ *P* values comparing the C-indices between models were estimated using a significance level of *P* < .05.

## Results

### Study Population

We identified 20 292 women with stage I to III breast cancer meeting the eligibility criteria (eFigure in Supplement 1 and Table 1). The median (IQR) age at chemotherapy initiation was 70 (67-74) years. In total, 4583 women (22.6%) had stage I, 10 935 (53.9%) had stage II, and 4774 (23.5%) had stage III breast cancer at diagnosis.

**Table 1.** Demographic and Clinical Characteristics of Women With Stage I to III Breast Cancer Diagnosed Between 2004 and 2017 in the Surveillance, Epidemiology, and End Results–Medicare Linked Database, Stratified by Claims-Based Frailty Trajectory During the Year Following Initiation of Adjuvant Chemotherapy

Characteristic	Patients, No. (%)^a^						
	Overall (N = 20 292)	Claims-based frailty trajectory					
		Robust (n = 16 120)	Resilient low-to-medium (n = 3028)	Resilient medium-to-high (n = 231)	Nonresilient low-to-medium (n = 665)	Nonresilient low-to-high (n = 149)	Nonresilient high (n = 99)
Demographics							
Age, median (IQR), y	70 (67-74)	70 (67-73)	73 (69-78)	74 (69-79)	74 (70-79)	74 (70-79)	74 (69-78)
Race							
American Indian or Alaska Native	81 (0.4)	32 (0.2)	37 (1.2)	NR^b^	NR^b^	NR^b^	NR^b^
Asian or Pacific Islander	907 (4.5)	418 (2.6)	418 (13.9)	NR^b^	NR^b^	NR^b^	NR^b^
Black	1643 (8.1)	1089 (6.8)	355 (11.8)	43 (18.7)	110 (16.6)	25 (16.8)	21 (21.2)
White	17 622 (87.0)	14 559 (90.4)	2205 (73.1)	171 (74.3)	499 (75.4)	119 (79.9)	69 (69.7)
Ethnicity							
Hispanic	1177 (5.8)	1039 (6.4)	81 (2.7)	15 (6.5)	35 (5.3)	NR^b^	NR^b^
Non-Hispanic	19 115 (94.2)	15 081 (93.6)	2947 (97.3)	216 (93.5)	630 (94.7)	NR^b^	NR^b^
Census region							
Northeast	3967 (19.5)	3259 (20.2)	526 (17.4)	39 (16.9)	102 (15.3)	NR^b^	NR^b^
West	8527 (42.0)	6696 (41.5)	1346 (44.5)	101 (43.7)	283 (42.6)	61 (40.9)	40 (40.4)
Midwest	3813 (18.8)	3007 (18.7)	571 (18.9)	52 (22.5)	134 (20.2)	NR^b^	NR^b^
South	3985 (19.6)	3158 (19.6)	585 (19.3)	39 (16.9)	146 (22.0)	28 (18.8)	29 (29.3)
Clinical characteristics							
Stage at diagnosis							
I	4583 (22.6)	3851 (23.9)	557 (18.4)	34 (14.7)	108 (16.2)	18 (12.1)	15 (15.2)
II	10 935 (53.9)	8745 (54.2)	1602 (52.9)	122 (52.8)	344 (51.7)	76 (51.0)	46 (46.5)
III	4774 (23.5)	3524 (21.9)	869 (28.7)	75 (32.5)	213 (32.0)	55 (36.9)	38 (38.4)
Type of surgery							
Mastectomy	8090 (39.9)	6189 (38.4)	1365 (45.1)	125 (54.1)	294 (44.2)	61 (40.9)	56 (56.6)
Breast-conserving therapy	12 202 (60.1)	9931 (61.6)	1663 (54.9)	106 (45.9)	371 (55.8)	88 (59.1)	43 (43.4)
T stage, No.							
T1	8501 (42.0)	7088 (44.1)	1061 (35.1)	73 (31.7)	210 (31.6)	43 (28.9)	26 (26.5)
T2	9730 (48.1)	7532 (46.8)	1567 (51.9)	127 (55.2)	363 (54.6)	92 (61.7)	49 (50.0)
T3	1425 (7.0)	1066 (6.6)	250 (8.3)	NR^b^	62 (9.3)	NR^b^	NR^b^
T4	583 (2.9)	391 (2.4)	142 (4.7)	NR^b^	30 (4.5)	NR^b^	NR^b^
N stage							
N0	8901 (43.9)	7210 (44.8)	1268 (42.0)	83 (35.9)	260 (39.1)	43 (28.9)	37 (37.4)
N1	7367 (36.3)	5927 (36.8)	1047 (34.7)	86 (37.2)	220 (33.1)	55 (36.9)	32 (32.3)
N2	2625 (13.0)	1972 (12.2)	460 (15.2)	38 (16.5)	106 (15.9)	32 (21.5)	17 (17.2)
N3	1377 (6.8)	997 (6.2)	245 (8.1)	24 (10.4)	79 (11.9)	19 (12.8)	13 (13.1)
Tumor grade							
Well differentiated	1705 (8.6)	1414 (9.0)	209 (7.1)	NR^b^	NR^b^	NR^b^	NR^b^
Moderately differentiated	7775 (39.4)	6392 (40.8)	1024 (34.7)	79 (34.8)	205 (31.6)	50 (34.2)	25 (26.0)
Poorly differentiated	10 109 (51.2)	7748 (49.5)	1694 (57.4)	128 (56.4)	396 (61.0)	86 (58.9)	57 (59.4)
Undifferentiated	143 (0.7)	109 (0.7)	24 (0.8)	NR^b^	NR^b^	NR^b^	NR^b^
Subtype^c^							
HR-positive and HER2-positive	2156 (18.0)	1652 (17.4)	379 (20.7)	NR^b^	75 (18.5)	NR^b^	16 (28.6)
HR-positive and HER2-negative	6232 (52.1)	5114 (54.0)	818 (44.7)	61 (44.5)	178 (43.8)	34 (48.6)	27 (48.2)
HR-negative and HER2-positive	912 (7.6)	646 (6.8)	186 (10.2)	NR^b^	59 (14.5)	NR^b^	NR^b^
HR-negative and HER2-negative	2673 (22.3)	2060 (21.7)	449 (24.5)	43 (31.4)	94 (23.2)	NR^b^	NR^b^
Gagne Combined Comorbidity score^d^							
≤0	7405 (36.5)	6817 (42.3)	531 (17.5)	15 (6.5)	39 (5.9)	NR^b^	NR^b^
1	4302 (21.2)	3777 (23.4)	458 (15.1)	15 (6.5)	44 (6.6)	NR^b^	NR^b^
2	2965 (14.6)	2385 (14.8)	479 (15.8)	15 (6.5)	65 (9.8)	NR^b^	NR^b^
≥3	5620 (27.7)	3141 (19.5)	1560 (51.5)	186 (80.5)	517 (77.7)	130 (87.2)	86 (86.9)

Abbreviations: HER2, human epidermal growth factor receptor 2; HR, hormone receptor; NR, not reported.

^a^ Values may not sum to totals due to missing data: 39 individuals had unknown or missing information on race, 53 had missing tumor size, 22 had missing lymph node involvement, 560 had missing tumor grade, and 8319 had missing tumor subtype.

^b^ Cell sizes less than 11 are suppressed.

^c^ HER2 status is only reliably captured in Surveillance, Epidemiology, and End Results–Medicare after 2010. Individuals who received a diagnosis before 2010 are assigned missing subtype.

^d^ The Gagne Combined Comorbidity score was assessed using claims between chemotherapy initiation and the landmark (360 days following initiation). Cancer and metastatic cancer were removed when calculating the Gagne Combined Comorbidity score.

### Trajectory Patterns

**Figure 2.**
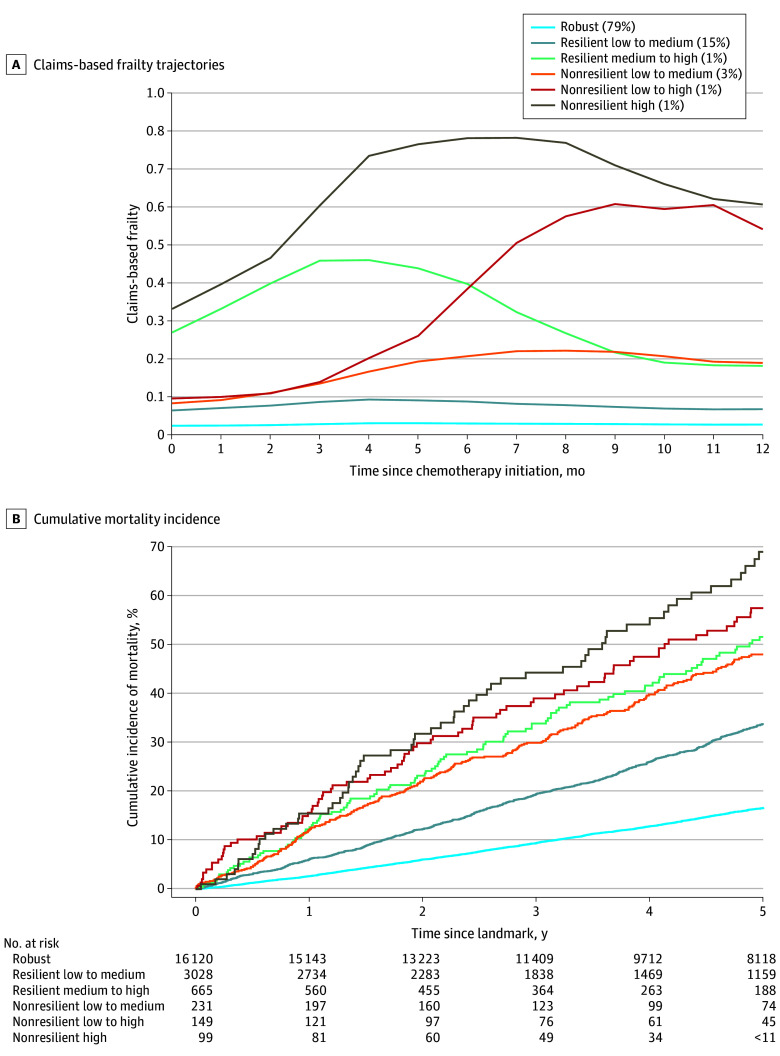
Frailty Trajectories Following Adjuvant Chemotherapy and 5-Year Mortality in Women With Breast Cancer A, Claims-based frailty trajectories during the year following adjuvant chemotherapy initiation (adapted from Duchesneau et al^10^). The claims-based frailty index has no units. Percentages in key show proportion of participants in each trajectory group. B, Five-year mortality by claims-based frailty trajectory among women with stage I to III breast cancer diagnosed between 2004 and 2017 in the Surveillance, Epidemiology, and End Results–Medicare linked database. Five-year risks were 17% (risk difference [RD], 0 [reference]) for the robust trajectory, 34% (RD, 17; 95% CI, 15-19) for the resilient low-to-medium trajectory, 52% (RD, 31; 95% CI, 27-36) for the resilient medium-to-high trajectory, 48% (RD, 41; 95% CI, 32-49) for the nonresilient low-to-medium trajectory, 58% (RD, 35; 95% CI, 28-43) for the nonresilient low-to-high trajectory, and 69% (RD, 52; 95% CI, 42-63) for the nonresilient high trajectory.

Most women had a robust frailty trajectory following chemotherapy initiation (16 120 women [79.4%]), 3259 (16.1%) had 1 of the 2 resilient trajectories, and 913 (4.5%) had 1 of the nonresilient trajectories (Figure 2A). Women with nonresilient frailty trajectories were older, had higher disease stage, and more comorbidities than those with robust or resilient trajectories (Table 1 and eTable 2 in Supplement 1).

Among women with robust or resilient trajectories, the frailty indicators with the largest absolute change in prevalence between chemotherapy initiation and the landmark were cancer screening (58.2% to 35.3%), lipid abnormalities (64.4% to 54.6%), and arthritis or joint conditions (43.5% to 36.4%) (eTable 3 in Supplement 1). Women with nonresilient trajectories showed larger changes. Prevalence of cancer screening (45.0% to 20.5%) and lipid abnormalities (65.2% to 54.8%) decreased, whereas weakness (11.8% to 33.7%), wheelchair use (5.1% to 22.7%), and ambulance or life support use (13.9% to 31.3%) increased.

### Frailty Trajectories and Mortality

Twenty-one percent of women died during the 5-year follow-up. Five-year mortality was higher in women belonging to the 3 nonresilient trajectories compared with those belonging to the 3 resilient trajectories (52.1% vs 20.3%; difference, 31.8%; 95% CI, 29.0%-36.2%). Women with a robust frailty trajectory had the lowest 5-year mortality (16.6%), followed by those belonging to the resilient low-to-medium trajectory (33.8%) (Figure 2B). Women with a resilient medium-to-high or a nonresilient low-to-medium frailty trajectory had similar 5-year mortality (51.6% and 48.0%, respectively), despite having different mean baseline claims-based frailty scores (0.27 and 0.08) that converged toward the landmark (0.18 and 0.19). Women with a nonresilient low-to-high trajectory and those with a nonresilient high trajectory had the highest 5-year mortality (57.5% and 69.0%, respectively).

### Subgroup Analyses

**Figure 3.**
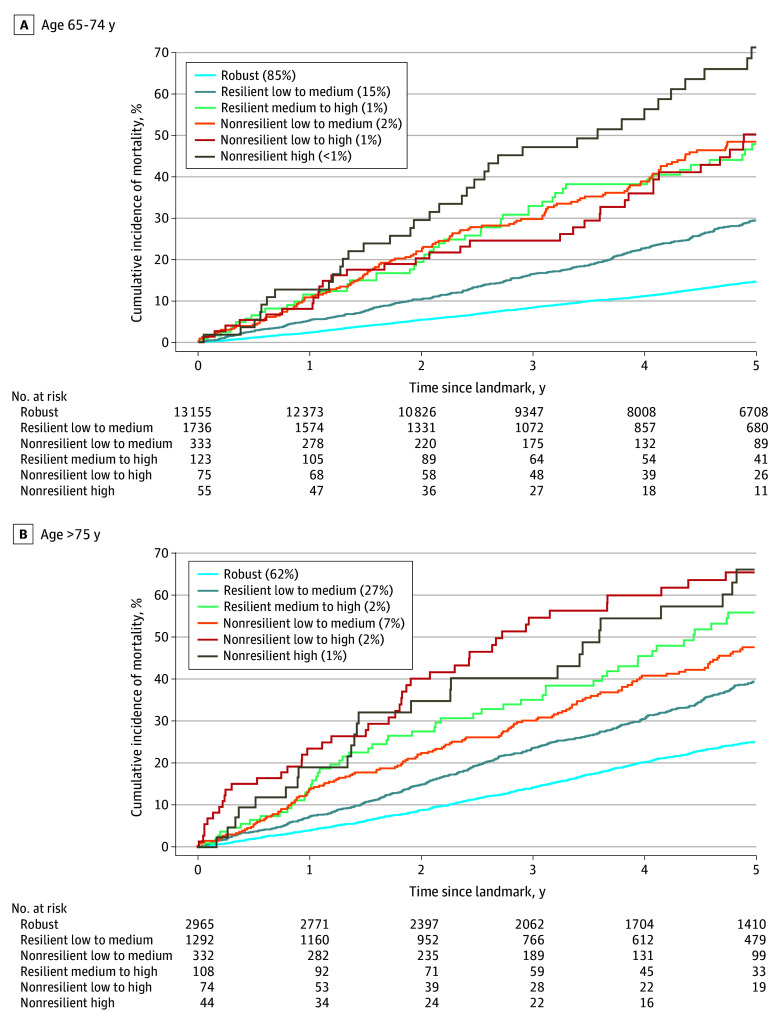
Five-Year Mortality in Women With Breast Cancer, by Frailty Trajectory and Age Percentages in keys show proportion of participants in each trajectory groups. Five-year mortality by claims-based frailty trajectory among women with stage I to III breast cancer diagnosed between 2004 to 2017 in the Surveillance, Epidemiology, and End Results–Medicare linked database, stratified by age at treatment initiation. For women aged 65 to 74 years (A), 5-year risks were 15% (risk difference [RD], 0 [reference]) for the robust trajectory, 29% (RD, 15; 95% CI, 12-17) for the resilient low-to-medium trajectory, 48% (RD, 34; 95% CI, 27-40) for the resilient medium-to-high trajectory, 48% (RD, 36; 95% CI, 22-48) for the nonresilient low-to-medium trajectory, 50% (RD, 33; 95% CI, 23-43) for the nonresilient low-to-high trajectory, and 71% (RD, 57; 95% CI, 43-70) for the nonresilient high trajectory. For women aged 75 years and older (B), 5-year risks were 25% (RD, 0 [reference]) for the robust trajectory, 39% (RD, 14; 95% CI, 11-18) for the resilient low-to-medium trajectory, 56% (RD, 23; 95% CI, 16-29) for the resilient medium-to-high trajectory, 48% (RD, 40; 95% CI, 28-52) for the nonresilient low-to-medium trajectory, 65% (RD, 31; 95% CI, 20-41) for the nonresilient low-to-high trajectory, and 66% (RD, 41; 95% CI, 25-57) for the nonresilient high trajectory.

Five-year mortality was higher in women aged 75 years and older compared with those aged 64 to 74 years (32% vs 18%; difference, 14%; 95% CI, 13%-16%). Generally, mortality increased with worsening frailty trajectories across age groups (Figure 3). In younger women (aged 64-74 years), mortality ranged from 15% in the robust trajectory to 71% in the nonresilient high trajectory. Similar trends were observed in women aged 75 years and older. However, women with resilient medium-to-high trajectories had higher mortality compared with those with nonresilient low-to-medium trajectories (56% vs 48%; difference, 9%; 95% CI, −6% to 26%; not statistically significant).

Five-year mortality increased with higher cancer stage among women with resilient and nonresilient trajectories (eTable 4 in Supplement 1). The difference in 5-year mortality risk comparing nonresilient vs resilient clusters was similar by cancer stage (stage I risk difference [RD], 29%; 95% CI, 18%-40%; stage II RD, 32%; 95% CI, 26%-37%; stage III RD, 30%; 95% CI, 24%-36%). Five-year mortality varied across subtypes: HR-positive and HER2-positive, 15%; HR-positive and HER2-negative, 17%; HR-negative and HER2-positive, 17%; and triple-negative, 22%. In all cancer subtype subgroups, nonresilient frailty trajectories were associated with increased mortality (eTable 4 in Supplement 1). The RDs varied by subtype, with HR-positive and HER2-positive having the largest difference (RD, 42%; 95% CI, 29%-56%) and triple-negative having the smallest (RD, 21%; 95% CI, 11%-31%).

### Frailty Trajectories and Mortality Predictions

In our Cox proportional hazards models, we found that the claims-based frailty trajectories were predictive of 5-year mortality among older women with stage I to III breast cancer (Table 2). The C-index for the model including patient characteristics and baseline frailty only (model 1) was 0.699 (95% CI, 0.690-0.708). Prediction was slightly improved in the model including patient characteristics, baseline frailty, and the frailty trajectories (model 2 C-index, 0.706; 95% CI, 0.697-0.715; *P* = .009 for difference vs model 1) and in the ad hoc model that included the patient characteristics, baseline frailty score, and the final frailty score calculated at the end of the landmark period (model 3 C-index, 0.705; 95% CI, 0.696-0.714; *P* = .008 for difference vs model 1).

**Table 2.** Cox Proportional Hazards Models Comparing Prediction of 5-Year Mortality Using Baseline and Claims-Based Frailty Trajectories in Women With Stage I to III Breast Cancer Diagnosed Between 2004 and 2017 in the Surveillance, Epidemiology, and End Results–Medicare Linked Database^a^

Variable	HR (95% CI)		
	Model 1	Model 2	Model 3^b^
Claims-based frailty at time of chemotherapy initiation	5.67 (4.13-7.78)	2.19 (1.38-3.49)	2.33 (1.62-3.35)
Claims-based frailty at end of landmark window (360 d following chemotherapy initiation)^b^	NA	NA	4.44 (3.44-5.74)
Claims-based frailty trajectory group during year following chemotherapy initiation			
Robust	NA	1 [Reference]	NA
Resilient low-to-medium	NA	1.52 (1.39-1.67)	NA
Resilient medium-to-high	NA	2.05 (1.78-2.37)	NA
Nonresilient low-to-medium	NA	1.94 (1.53-2.47)	NA
Nonresilient low-to-high	NA	2.43 (1.91-3.09)	NA
Nonresilient high	NA	2.25 (1.61-3.15)	NA
C-index (95% CI)	0.699 (0.690-0.708)	0.706 (0.697-0.715)	0.705 (0.696-0.714)
Difference in C-index (95% CI)	0 [Reference]	0.007 (0.005-0.009)	0.005 (0.004-0.007)
*P* value	Not applicable	.009	.008

Abbreviations: HR, hazard ratio; NA, not applicable.

^a^ All 3 models were adjusted for age at the time of chemotherapy initiation, race, Hispanic ethnicity, type of breast surgery (mastectomy or breast conserving therapy), stage at diagnosis (I, II, III), census region, year of diagnosis, and the Gagne Combined Comorbidity score.

^b^ Model 3 was an ad hoc analysis based on preliminary findings that 5-year mortality was similar across trajectory clusters that had similar average frailty score measured at the end of the landmark period.

## Discussion

In this cohort analysis of a large population-based sample of older women with stage I to III breast cancer, we found that claims-based frailty trajectories during the year following adjuvant chemotherapy initiation were associated with 5-year mortality. Women with robust or resilient frailty trajectories had better survival than their counterparts who experienced increasing or consistently high frailty. Women who experienced moderate or rapid increases in claims-based frailty (the nonresilient low-to-medium and nonresilient low-to-high clusters) had worse survival than those belonging to the resilient low-to-medium trajectory cluster, despite having similar mean claims-based frailty at the time of chemotherapy initiation. These nonresilient clusters also had similar or worse survival compared with women who had consistently moderate frailty (resilient medium-to-high cluster). Women with consistently high frailty (nonresilient high cluster) had the highest 5-year mortality.

Despite the associations between frailty trajectories and 5-year mortality, the C-indices from our Cox proportional hazards models suggested that longitudinal frailty trajectory clusters only marginally improved mortality predictions compared with a model that only included the baseline frailty score and covariates. Although the discrepancy between our measures of association and model discrimination appears contradictory, this phenomenon has been reported in other biomedical literature.^27,28^ Measures of association, like the results from our Kaplan-Meier analyses, are useful for drawing inferences to an overall population. Alternatively, measures of model discrimination, such as the C-index, are useful for evaluating the prognostic strength of a model for correctly classifying individual risks. Although women with robust or resilient frailty trajectories had lower mortality risk, these groups encompassed 94% of the cohort, and thus still accounted for most deaths (89%). Taken together, our results suggest that although claims-based frailty trajectories are useful for understanding differences in population average risks, they may not be as useful in understanding individual prognosis. It is possible that the frailty trajectories may provide greater prognostic utility for certain subgroups who are at higher risk of mortality (eg, high baseline frailty or comorbidity burden), which should be investigated in future work. In addition, future research should assess whether claims-based frailty trajectories are predictive of other outcomes that are important to clinicians and patients, such as falls, hospitalizations, skilled nursing facility admissions, and quality of life.

The association between frailty measured at the time of breast cancer diagnosis or treatment initiation and mortality is well understood.^5,29^ However, few studies have assessed whether changes in frailty during cancer treatment are associated with long-term survival. Prior research^30,31,32^ has shown that changes in physical function, a measure of physiological capacity that is related to but distinct from frailty, are associated with prognosis following cancer diagnosis and treatment. In a Women’s Health Initiative study,^30^ postmenopausal women who experienced a decline in self-reported physical function within 1 year of a cancer diagnosis had worse all-cause and cancer-specific mortality than those without functional decline. Similarly, a multisite prospective study^31^ of older women with early-stage breast cancer found that women who experienced large and persistent declines in self-reported physical function following diagnosis had higher 10-year mortality than their counterparts. In a broader cohort of 439 older adults with a malignant tumor who initiated chemotherapy, older adults who experienced functional decline following chemotherapy initiation were more likely to die than those without functional decline.^32^ Associations between frailty trajectories and mortality have been observed in general populations of older adults.^33^ Our findings contribute to this literature by measuring granular frailty changes in monthly time intervals following adjuvant chemotherapy initiation, when chemotherapy toxic effects and cancer sequelae may contribute to frailty progression and associated outcomes.

Although the proportion of women in our study who experienced nonresilient frailty trajectories following chemotherapy initiation was small (4.5%), these women may benefit from interventions to reduce frailty progression, such as nutritional, physical activity, and supportive care interventions.^34,35,36,37,38^ Our prior research and others have found that Black women, those with comorbid conditions, and those with worse performance status are prone to experiencing nonresilient frailty trajectories during cancer treatment.^10,39^ Research is needed to understand whether changes in frailty are driven by modifiable risk factors, such as incident cardiotoxic effects that could be prevented or treated,^40^ vs systemic physiologic changes that may be less amenable to treatment.

We identified claims-based frailty trajectories using monthly assessments and K-means longitudinal clustering methods, which are useful for identifying patterns in repeated measures. A strength of these methods is their simplicity and efficiency in handling large datasets and multiple time points. However, although results from K-means clustering analyses are useful for hypothesis generation, they should be considered exploratory rather than definitive. Additional research using other longitudinal methods is necessary to validate and extend our findings. Future research should investigate the utility of assessing frailty and other aging-related impairments at multiple time points during the cancer survivorship continuum using clinical tools, such as comprehensive geriatric assessment or electronic health record–derived frailty indices,^41,42^ to improve long-term outcomes in older patients with cancer.

A strength of our analysis is the use of the population-based SEER-Medicare database, which includes a large and diverse sample of older women with breast cancer. These data may better reflect older women with breast cancer seen in routine oncology care compared with clinical trials.^43,44^ Although clinical measures of frailty, such as the frailty phenotype,^45^ are not available in claims data, claims-based frailty indices such as the Faurot frailty index have been shown to discriminate frailty well (using the frailty phenotype and deficit accumulation frailty index as reference standards).^17,46,47^ These measures have been applied in many studies focused on evaluating cancer outcomes and therapeutics.^48,49,50^ A recent study^51^ also found that older adults who experienced a 3-year worsening in the frailty phenotype experienced a concurrent average increase in the Faurot frailty index, suggesting the measure is able to identify clinically meaningful worsening of frailty over time.

### Limitations

This study has limitations that should be mentioned. We used a landmark design to assign women to claims-based frailty trajectories following chemotherapy initiation. Although this method avoids many time-related biases,^14^ it required exclusion of individuals who died or disenrolled from Medicare during the year following treatment initiation. Therefore, our results likely do not generalize to the most ill and frail women, who are most likely to die during this period. Our results also do not generalize to women who are frail and considered unfit for chemotherapy by their physicians. Owing to small sample sizes, we consolidated frailty trajectories into 2 clusters (resilient and nonresilient) for subgroup analyses by cancer stage and subtype, potentially masking important heterogeneity within clusters. In addition, our analysis focused on associations between frailty trajectories and mortality; however, frailty is also associated with other outcomes, including treatment discontinuation, chemotherapy toxic effects, hospitalizations, and poor quality of life, which may be important mediators on the path between frailty and mortality.^4,5,6^

## Conclusions

In this cohort study of older women with stage I to III breast cancer, frailty changes following adjuvant chemotherapy initiation were associated with long-term survival. Future research is needed to understand whether improved frailty monitoring and interventions for older women with breast cancer during cancer treatment can improve outcomes.

## Notes

Supplement 1. **eTable 1. **Diagnosis and procedure codes to identify the components of the Faurot frailty index (adapted from Duchesneau et al. 2023)1
eReference.
**eFigure.** Consort diagram **eTable 2.** Prevalence of the conditions of the Gagne comorbidity index during the year following chemotherapy initiation among women with stage I-III breast cancer in the SEER-Medicare linked database, stratified by claims-based frailty trajectories **eTable 3.** Prevalence of the indicators of the Faurot frailty index at chemotherapy initiation and the landmark among women with stage I-III breast cancer in the SEER-Medicare linked database, stratified by resilient and non-resilient trajectories **eTable 4.** Cumulative incidence (risk) of five-year mortality by resilient vs. non-resilient claims-based frailty trajectories among women with stage I-III breast cancer in the SEER-Medicare linked database, stratified by tumor stage and subtype

Supplement 2.
Data Sharing Statement
