# Supplementary material for: Frailty Trajectories Following Adjuvant Chemotherapy and Mortality in Older Women With Breast Cancer
Source: JAMA Netw Open. 2025 Mar 12;8(3):e250614. doi: 10.1001/jamanetworkopen.2025.0614 (PMC11904708; doi:10.1001/jamanetworkopen.2025.0614)
Supplement: Supplement 1. — eTable 1. Diagnosis and procedure codes to identify the components of the Faurot frailty index (adapted from Duchesneau et al. 2023)1 eReference. eFigure. Consort diagram eTable 2. Prevalence of the conditions of the Gagne comorbidity index during the year following chemotherapy initiation among women with stage I-III breast cancer in the SEER-Medicare linked database, stratified by claims-based frailty trajectories eTable 3. Prevalence of the indicators of the Faurot frailty index at chemotherapy initiation and the landmark among women with stage I-III breast cancer in the SEER-Medicare linked database, stratified by resilient and non-resilient trajectories eTable 4. Cumulative incidence (risk) of five-year mortality by resilient vs. non-resilient claims-based frailty trajectories among women with stage I-III breast cancer in the SEER-Medicare linked database, stratified by tumor stage and subtype [file jamanetwopen-e250614-s001.pdf]

Supplemental Online Content

Duchesneau ED, Kim DH, Stürmer T, et al. Frailty trajectories following adjuvant chemotherapy and mortality in older women with breast cancer. *JAMA Netw Open*. 2025;8(3):e250614. doi:10.1001/jamanetworkopen.2025.0614

eTable 1. Diagnosis and procedure codes to identify the components of the Faurot frailty index (adapted from Duchesneau et al. 2023)[1]

eReference

eFigure. Consort diagram

eTable 2. Prevalence of the conditions of the Gagne comorbidity index during the year following chemotherapy initiation among women with stage I-III breast cancer in the SEER-Medicare linked database, stratified by claims-based frailty trajectories

eTable 3. Prevalence of the indicators of the Faurot frailty index at chemotherapy initiation and the landmark among women with stage I-III breast cancer in the SEER-Medicare linked database, stratified by resilient and non-resilient trajectories

eTable 4. Cumulative incidence (risk) of five-year mortality by resilient vs. non-resilient claims-based frailty trajectories among women with stage I-III breast cancer in the SEER-Medicare linked database, stratified by tumor stage and subtype

This supplemental material has been provided by the authors to give readers additional information about their work.

© 2025 Duchesneau ED et al. *JAMA Network Open*.

eTable 1. Diagnosis and procedure codes to identify the components of the Faurot frailty index (adapted from Duchesneau et al. 2023)[1]

Indicator	ICD-9-CM	ICD-10-CM [a]	HCPCS/CPT
Intercept	N/A	N/A	N/A
Age (centered: age-65)	N/A	N/A	N/A
Age-centered squared	N/A	N/A	N/A
Female gender	N/A	N/A	N/A
Non-Hispanic Black race [b]	N/A	N/A	N/A
Hispanic/Latino	N/A	N/A	N/A
Non-Hispanic other race	N/A	N/A	N/A
Cancer screening	V76.	Z12.	
Lipid abnormality	272.	E71.30, E75.21, E75.22, E75.24, E75.3, E75.5, E75.6, E77.0, E78.0, E78.1, E78.2, E78.3, E78.4, E78.5, E78.6, E78.70, E78.79, E78.8, E78.9, E88.1, E88.89	
Vertigo	386., 438.85, 780.4	H81., H82., H83.0, H83.1, H83.2, R42.	
Arthritis and joint conditions	710., 711., 712., 714., 716.5, 716.6, 716.8, 716.9, 718., 719.0, 719.1, 719.4, 719.5, 719.9, 725.	M00., M01., M02.1, M02.3, M02.8, M04.2, M04.8, M04.9, M05., M06.0, M06.1, M06.3, M06.4, M06.8, M06.9, M07.6, M08.0, M08.2, M08.3, M08.4, M08.8, M08.9, M11.1, M11.2, M11.8, M11.9, M12.0, M12.8, M12.9, M13.0, M13.1, M15., M16., M17., M18., M19.0, M19.1, M19.2, M19.9, M22.0, M22.1, M23.5, M24.0, M24.1, M24.3, M24.4, M24.5, M24.6, M24.7, M24.8, M24.9, M25.0, M25.2, M25.3, M25.4, M25.5, M25.6, M25.9, M32.10, M32.12, M32.13, M32.14, M32.15, M32.19, M32.8, M32.9, M33., M34., M35.00, M35.01, M35.02, M35.03, M35.04, M35.09, M35.1, M35.2, M35.3, M35.5, M35.8, M35.9, M36.8, M43.3, M43.4, M43.5X2, M43.5X3, M43.5X4, M43.5X5, M43.5X6, M43.5X7, M43.5X8, M43.5X9, M79.646	

© 2025 Duchesneau ED et al. *JAMA Network Open*.

Indicator	ICD-9-CM	ICD-10-CM [a]	HCPCS/CPT
Bladder dysfunction	596.5, 599.6, 788.2, 788.3	N13., N31., N32., N36., N39.3, N39.4, N39.8, N39.9, R32., R33., R39.14, R39.81	
Podiatric care	681.1, 700., 703.	L02.61, L03.03, L03.04, L60., L62., L84.	
Heart failure	425., 428., 429.0, 429.1, 429.3, 429.4	I09.81, I11.0, I13.0, I13.2, I25.5, I42., I43., I50., I51.4, I51.5, I51.7, I97.0, I97.11, I97.120, I97.13, I97.19	
Psychiatric illness	290., 291., 292., 293., 294., 295., 296., 297., 298., 299., 300.0, 310., 311.	F01., F02., F03., F04., F05., F06., F07., F09., F10.13, F10.14, F10.15, F10.180, F10.182, F10.188, F10.23, F10.24, F10.25, F10.26, F10.27, F10.280, F10.282, F10.93, F10.94, F10.95, F10.96, F10.97, F10.980, F10.982, F11.13, F11.14, F11.15, F11.182, F11.23, F11.24, F11.25, F11.282, F11.93, F11.94, F11.95, F11.982, F12.13, F12.15, F12.180, F12.23, F12.25, F12.280, F12.93, F12.95, F12.980, F13.13, F13.14, F13.15, F13.180, F13.182, F13.23, F13.24, F13.25, F13.26, F13.27, F13.280, F13.282, F13.93, F13.94, F13.95, F13.96, F13.97, F13.980, F13.982, F14.13, F14.14, F14.15, F14.180, F14.182, F14.23, F14.24, F14.25, F14.280, F14.282, F14.93, F14.94, F14.95, F14.980, F14.982, F15.13, F15.14, F15.15, F15.180, F15.182, F15.23, F15.24, F15.25, F15.280	
Rehabilitation care	V57.1, V57.21, V57.3, V57.8, V57.9	Z51.89	97110, 97161, 97162, 97116, 97535, 92507, 97164, 97012, 97112, 97530, 97113
Home oxygen	N/A	N/A	E1390, E1391, E1392, E0431, E0433, E0434, E0435, E0439, E0441, E0442, E0443
Hypotension or shock	458., 785.5, 958.4, 998.0	I95., R57., R65.2, T79.4, T81.1	
Ambulance transport	N/A	N/A	A0426, A0427, A0428, A0429, A0999
Stroke/brain injury	348., 349.82, 430., 431., 432., 433.01, 433.11, 433.21, 433.31, 433.91, 434.01, 434.11, 434.91, 436., 852., 853., 854.	G92., G93.1, G93.4, G93.5, G93.6, G93.89, G93.9, I60., I61., I62., I63.00, I63.01, I63.02, I63.03, I63.10, I63.11,	

© 2025 Duchesneau ED et al. *JAMA Network Open*.

Indicator	ICD-9-CM	ICD-10-CM [a]	HCPCS/CPT
		I63.12, I63.13, I63.2, I63.3, I63.4, I63.5, I63.6, I63.8, I63.9, I67.83, I67.89, S01.90, S06.1, S06.2, S06.30, S06.34, S06.35, S06.36, S06.4, S06.5, S06.6, S06.8, S06.9	
Dementias	290., 294., 331., 333.90, 333.92, 333.99, 438.0, 780.93, 797.	F01., F02., F03., F04., F06.8, G13.8, G21.0, G25.7, G25.89, G25.9, G26., G30., G31.0, G31.1, G31.83, G31.84, G31.85, G31.89, G31.9, G91.1, G91.2, G91.3, G91.8, G91.9, G94., I69.010, I69.011, I69.014, I69.015, I69.018, I69.019, I69.110, I69.111, I69.114, I69.115, I69.118, I69.119, I69.210, I69.211, I69.215, I69.218, I69.219, I69.310, I69.311, I69.314, I69.315, I69.318, I69.319, I69.810, I69.811, I69.814, I69.815, I69.818, I69.819, I69.91, R41.1, R41.2, R41.3, R41.81	
Parkinson’s Disease	332.	G20., G21.1, G21.2, G21.3, G21.4, G21.8, G21.9	
Weakness	728.2, 728.3, 728.87, 799.3, V49.84	M62.5, M62.81, M62.84, M62.89, R53.81, R54., Z74.0, Z74.01	
Skin ulcer (decubitus)	707.	L89., L97., L98.4	
Paralysis	342., 344., 438.2, 438.3, 438.4, 438.5, 781.4	G81., G82., G83.1, G83.2, G83.3, G83.5, G83.89, G83.9, I69.03, I69.04, I69.05, I69.06, I69.13, I69.14, I69.15, I69.16, I69.23, I69.24, I69.25, I69.26, I69.33, I69.34, I69.35, I69.36, I69.83, I69.84, I69.85, I69.86, I69.93, I69.94, I69.95, I69.96, R29.5	
Wheelchair	N/A	N/A	E1050, E1060, E1070, E1083, E1084, E1085, E1086, E1087, E1088, E1089, E1090, E1091, E1092, E1093, E1100, E1110, E1140, E1150, E1160, E1161, E1170, K0001, K0002, K0003, K0004, K0005, K0006, K0007, K0008, K0009
Home hospital bed	N/A	N/A	E0250, E0251, E0255, E0256, E0260, E0261, E0265, E0266, E0270, E0290, E0291, E0292, E0293, E0294, E0295,

© 2025 Duchesneau ED et al. *JAMA Network Open*.

Indicator	ICD-9-CM	ICD-10-CM [a]	HCPCS/CPT
			E0296, E0297, E0301, E0302, E0303, E0304, E0316

Abbreviations: CPT=Current Procedural Terminology, HCPCS=Healthcare Common Procedure Coding System; ICD-9-CM=International Classification of Diseases, 9th Revision, Clinical Modification; ICD-10-CM: International Classification of Diseases, 10th Revision, Clinical Modification; N/A=not applicable.

eReference

1. Duchesneau ED, Shmuel S, Faurot KR, et al. Translation of a claims-based frailty index from International Classification of Diseases 9th Revision to 10th Revision. *Am J Epidemiol*. Jul 11 2023;doi:10.1093/aje/kwad151

© 2025 Duchesneau ED et al. *JAMA Network Open*.

eFigure. Consort diagram.

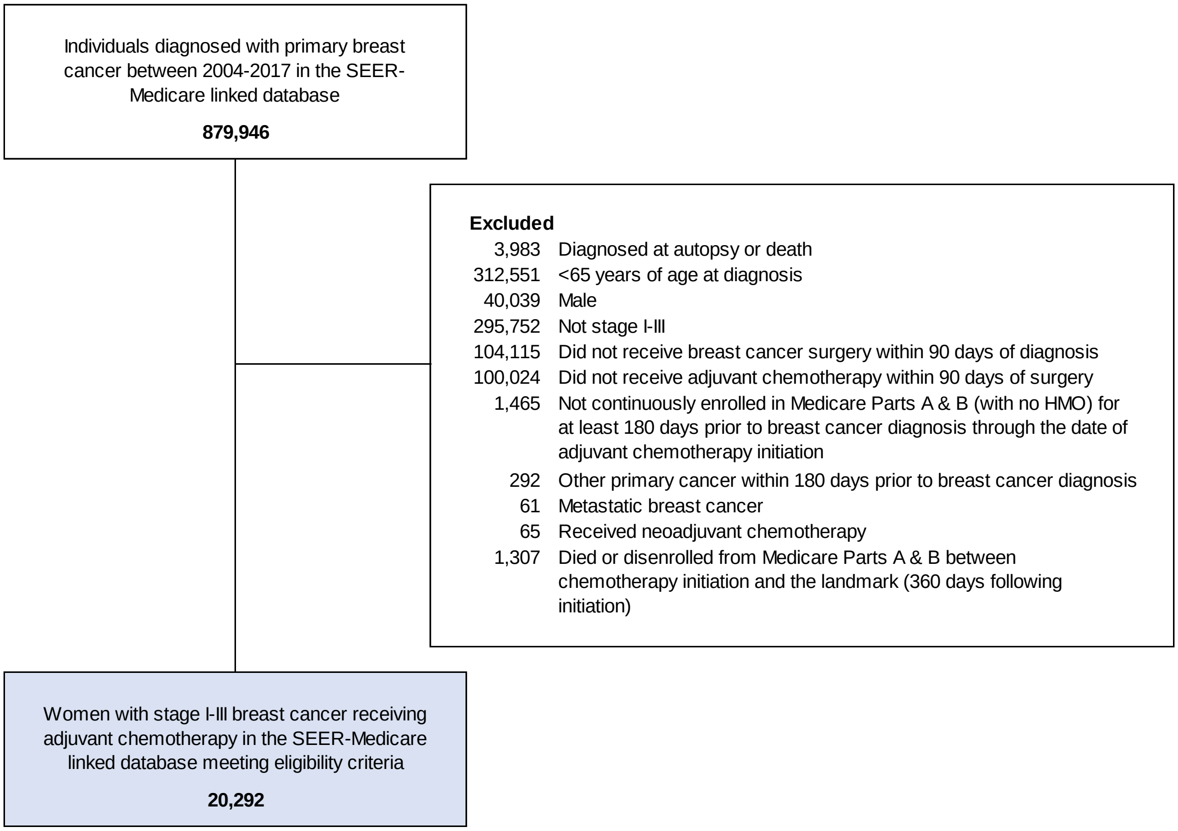

Abbreviations: HMO=health maintenance organization; SEER=Surveillance, Epidemiology & End Results.

© 2025 Duchesneau ED et al. *JAMA Network Open*.

eTable 2. Prevalence of the conditions of the Gagne comorbidity index during the year following chemotherapy initiation among women with stage I-III breast cancer in the SEER-Medicare linked database, stratified by claims-based frailty trajectories

		**Claims-based frailty trajectory, %**					
Comorbidity	% Overall N=20,292	Robust N=16,120	Resilient low/medium N=3,028	Resilient medium/high N=231	Non-resilient low-to-medium N=665	Non-resilient low-to-high N=149	Non-resilient high N=99
Alcohol abuse	0.7	0.5	1.2	*	*	*	*
Deficiency anemias	41.3	37.0	53.0	68.0	68.6	83.2	79.8
Cardiac arrhythmias	21.1	17.3	31.4	45.9	43.5	58.4	63.6
Congestive heart failure	20.1	14.3	36.2	59.7	54.7	64.4	67.7
Coagulopathy	10.8	8.7	16.2	22.9	25.0	26.8	27.3
Complicated diabetes	10.4	8.2	16.0	25.5	26.2	30.2	29.3
Chronic pulmonary disease	21.3	17.1	33.3	51.5	45.4	53.0	62.6
Dementia	1.7	0.3	4.1	15.6	12.5	16.8	33.3
Fluid and electrolyte disorders	36.7	31.9	50.9	68.0	64.4	75.8	74.7
Hemiplegia	0.8	0.1	1.0	10.4	4.5	19.5	32.3
HIV/AIDS	*	*	*	*	*	*	*
Hypertension	72.2	68.8	82.4	90.9	91.4	94.0	98.0
Liver disease	4.0	3.6	5.3	5.2	6.9	*	*
Psychosis	9.8	6.7	18.2	24.2	29.2	36.9	40.4
Pulmonary circulation disorders	1.7	1.1	3.3	6.9	5.0	*	*
Peripheral vascular disease	12.9	9.6	21.5	39.8	32.3	44.3	44.4
Renal failure	8.4	6.2	14.0	27.3	24.1	26.8	33.3
Weight loss	3.6	2.1	7.2	13.4	12.8	22.8	29.3

*Cell sizes <11 are suppressed.

© 2025 Duchesneau ED et al. *JAMA Network Open*.

eTable 3. Prevalence of the indicators of the Faurot frailty index at chemotherapy initiation and the landmark among women with stage I-III breast cancer in the SEER-Medicare linked database, stratified by resilient and non-resilient trajectories

	**Resilient N=19,379**		**Non-resilient N=913**	
	**Chemotherapy initiation**	**Landmark (360 days post-initiation)**	**Chemotherapy initiation**	**Landmark (360 days post-initiation)**
Ambulance/life support	3.5	3.5	13.9	**31.3**
Arthritis/joint conditions	43.5	**36.4**	62.2	64.5
Bladder dysfunction	4.7	4.1	12.5	**17.5**
Stroke/brain injury	2.5	2.4	10.4	**20.3**
Skin ulcer (decubitus)	1.3	1.4	3.9	**15.0**
Dementia	2.7	2.8	14.7	**26.6**
Heart failure	14.6	11.2	33.3	**40.7**
Home hospital bed	0.2	0.2	3.2	**11.6**
Hypotension/shock	1.9	1.7	3.0	**11.7**
Lipid abnormality	64.4	**54.6**	65.2	**54.8**
Home oxygen	1.6	2.2	11.4	**21.6**
Paralysis	0.6	0.3	4.7	**10.7**
Parkinson' disease	0.3	0.4	3.8	5.4
Podiatric care	4.7	7.1	16.2	**24.6**
Psychiatric diagnoses	22.1	**16.0**	41.5	**50.1**
Rehabilitation services	16.8	20.5	19.8	**27.7**
Cancer screening	58.2	**35.3**	45.0	**20.5**
Vertigo	6.2	6.8	12.0	13.1
Weakness	3.9	5.0	11.8	**33.7**
Wheelchair	0.4	0.5	5.1	**22.7**

Indicators for which the absolute change between chemotherapy initiation and the landmark was greater than five percentage points are bolded. The six claims-based frailty trajectories were consolidated into “resilient” and “non-resilient” trajectories due to sample size constraints.

© 2025 Duchesneau ED et al. *JAMA Network Open*.

eTable 4. Cumulative incidence (risk) of five-year mortality by resilient vs. non-resilient claims-based frailty trajectories among women with stage I-III breast cancer in the SEER-Medicare linked database, stratified by tumor stage and subtype

	**Resilient**		**Non-resilient**		
	**N**	**Risk (%)**	**N**	**Risk (%)**	**RD (95% CI)**
Cancer stage					
I	4,442	10.2	141	39.2	28.9 (18.2, 40.4)
II	10,469	17.2	466	48.8	31.6 (26.4, 36.8)
III	4,468	32.9	306	62.6	29.7 (23.5, 35.6)
Cancer subtype[a]					
HR+/HER2+	2,054	12.6	102	54.7	42.1 (28.6, 55.6)
HR+/HER2-	5,993	16.3	239	45.3	29.0 (21.3, 36.6)
HR-/HER2+	842	14.8	70	45.6	30.8 (15.9, 46.3)
HR-/HER2-	2,552	20.7	121	41.2	20.5 (10.9, 31.1)

Abbreviations: CI=confidence interval; HER2=human epidermal growth factor receptor 2; HR=hormone receptor; RD=risk difference; SEER=Surveillance, Epidemiology, and End Results.

[a] The subgroup analysis by tumor subtype only includes women diagnosed after 2010, since HER2 status was not captured in SEER-Medicare reliably before 2010.

© 2025 Duchesneau ED et al. *JAMA Network Open*.
