# Supplementary material for: Frailty Trajectories Following Adjuvant Chemotherapy and Mortality in Older Women With Breast Cancer
Source: JAMA Netw Open. 2025 Mar 12;8(3):e250614. doi: 10.1001/jamanetworkopen.2025.0614 (PMC11904708; doi:10.1001/jamanetworkopen.2025.0614)
Supplement: Supplement 2. — Data Sharing Statement [file jamanetwopen-e250614-s002.pdf]

Data Sharing Statement

Duchesneau. Frailty Trajectories Following Adjuvant Chemotherapy and Mortality in Older Women With Breast Cancer. *JAMA Netw Open*. Published March 12, 2025. doi:10.1001/jamanetworkopen.2025.0614

Data
Data available: No

Additional Information
Explanation for why data not available: This study used the linked Surveillance, Epidemiology, and End Results (SEER)-Medicare database. Data are available to researchers through a data use agreement: https://healthcaredelivery.cancer.gov/seermedicare/.
